# Association between chronic health conditions and severe acute respiratory syndrome in pregnant women: an exploratory study

**DOI:** 10.1590/1980-220X-REEUSP-2023-0336en

**Published:** 2024-11-22

**Authors:** José Cláudio Garcia Lira, Márcio Flávio Moura de Araújo, Flavia Paula Magalhães Monteiro, Roberto Wagner Júnior Freire de Freitas, Carla Regina de Sousa Teixeira, Floriacy Stabnow Santos, Ana Cristina Pereira de Jesus Costa, Marcelino Santos, Lívia Maia Pascoal

**Affiliations:** 1 Universidade Federal do Piauí, Departamento de Enfermagem, Floriano, PI, Brazil.; 2 Fundação Oswaldo Cruz, Eusébio, CE, Brazil.; 3 Universidade da Integração Internacional da Lusofonia Afro-Brasileira, Redenção, CE, Brazil.; 4 Universidade de São Paulo, Departamento de Enfermagem, Ribeirão Preto, SP, Brazil.; 5 Universidade Federal do Maranhão, Imperatriz, MA, Brazil.

**Keywords:** COVID-19, Severe Acute Respiratory Syndrome, Diabetes Mellitus, Pregnancy, COVID-19, Síndrome Respiratorio Agudo Grave, Diabetes Mellitus, Embarazo

## Abstract

**Objective:**

To analyze the association between chronic health conditions and severe acute respiratory syndrome in pregnant women.

**Method:**

Retrospective, exploratory study conducted with 1,152 pregnant women from all 27 states of Brazil who sought hospital treatment and were diagnosed with severe acute respiratory syndrome between 2020 and 2022. Public data from the Influenza Epidemiological Surveillance Information System (SIVEP-Gripe) of the Brazilian Ministry of Health were used. Nonparametric tests were performed in data interpretation.

**Results:**

The mortality rate of pregnant women due to severe acute respiratory syndrome in Brazil was 7%. Severe acute respiratory syndrome was statistically associated with the previous presence of diabetes (p = 0.023), neurological disease (p = 0.001), and drug use (p = 0.001). The epidemiological investigation of respiratory syndrome cases took longer in Black pregnant women (p = 0.012), unvaccinated women (p < 0.001) and women living in the north and south of the country (p = 0.011).

**Conclusion:**

Severe acute respiratory syndrome was more common in pregnant women with diabetes, neurological disease and drug users. However, these conditions did not lead to an increase in the number of deaths.

## RESUMEN

**Objetivo:**

Analizar la asociación entre condiciones crónicas de salud y síndrome respiratorio agudo severo en mujeres embarazadas.

**Método:**

Estudio exploratorio retrospectivo realizado con 1.152 mujeres embarazadas de los 27 estados de Brasil que buscaron tratamiento hospitalario y fueron diagnosticadas con síndrome respiratorio agudo severo entre 2020 y 2022. Se utilizaron datos públicos del Sistema de Información de Vigilancia Epidemiológica de la Influenza (SIVEP-Gripe) del Ministerio de Salud de Brasil. Se realizaron pruebas no paramétricas en la interpretación de los datos.

**Resultados:**

La tasa de mortalidad de mujeres embarazadas por síndrome respiratorio agudo severo en Brasil fue del 7%. El síndrome respiratorio agudo severo se asoció estadísticamente con la presencia previa de diabetes (p = 0,023), enfermedad neurológica (p = 0,001) y uso de drogas (p = 0,001). La investigación epidemiológica de los casos de síndrome respiratorio agudo severo fue más prolongada en las embarazadas de raza negra (p = 0,012), las no vacunadas (p < 0,001) y las residentes en el norte y sur del país (p = 0,011).

**Conclusión:**

El síndrome respiratorio agudo severo fue más frecuente en las embarazadas con diabetes, enfermedades neurológicas y usuarias de drogas. Sin embargo, estas condiciones no provocaron un aumento en el número de muertes.

## INTRODUCTION

Pregnant women are among the most vulnerable groups to Severe Acute Respiratory Syndromes (SARS) compared to the general population, regardless of the etiological agent^(1,2)^. The demand for prenatal care is intensified in the presence of the SARS-CoV-2 virus, which causes COVID-19, leading to an increase in therapeutic complexity and requiring even more urgent interventions^(3,4)^.

In Brazil, the maternal mortality rate due to COVID-19 increased in the initial months after the first cases of the disease appeared in the country, and was twice as high the following year^(5)^. Furthermore, evidence has shown that the number of hospitalizations, need for critical care, use of vasoactive substances and mechanical ventilation related to this infection appear to be higher among pregnant women. A higher rate of premature labor, preeclampsia and neonatal admission to intensive care units stand out among the associated consequences^(6, 7, 8)^.

The adverse effects are closely related to risk behaviors, such as substance use and abuse, excess weight, and chronic health conditions, such as diabetes^(6)^. In addition, the pregnancy-puerperal period has become the center of discussions in healthcare because it induces physiological changes and responses that increase the risk of respiratory, cardiovascular, neurological, and metabolic complications, especially due to the potential outcomes involved. Even so, data available on the specific characteristics of pregnant women that should receive greater attention from health professionals in hospitals and maternity wards worldwide are limited^(9, 10, 11, 12, 13)^.

Therefore, such information must be explored to gain a deeper understanding of the occurrence of SARS during pregnancy and related conditions for the development of healthcare. The objective of this study was to analyze the association between chronic health conditions and SARS in pregnant women.

## METHOD

### Study Design

This is a retrospective, multicenter, documentary study based on the analysis of data from the Influenza Epidemiological Surveillance Information System (Portuguese acronym: SIVEP-Gripe) of the Brazilian Ministry of Health from 2020 to 2022.

### Location, Population, and Selection Criteria

All pregnant women with SARS due to influenza or COVID-19 infection who sought hospital care between 2020 and 2022 in the 27 Brazilian states and the Federal District were included. Pregnant women had to have an epidemiological investigation form for SARS in a hospital environment to be included in the study. For epidemiological purposes, cases of SARS in pregnant women hospitalized with fever (even if reported), accompanied by cough or sore throat and who presented dyspnea or oxygen saturation (O_2_) <95%; or respiratory distress; or who died from SARS, regardless of hospitalization were considered.

### Sample Definition

The sample consisted of 1,152 pregnant women who had been hospitalized with a diagnosis of SARS. Participants were included based on the analysis of all complete records reported in the SIVEP-Gripe system in the aforementioned time frame.

### Data Collection

Data were collected from the SIVEP-Gripe system in January 2023 with the support of a form containing 41 questions that was previously applied by health professionals from epidemiological surveillance of different health services (basic health units, outpatient clinics, hospitals and rehabilitation units). Sociodemographic questions containing information about age, age group, gestational age, skin color, education, region and notification of hospital infection were considered, as well as questions related to comorbidities or pre-existing health factors, namely: a) cardiovascular diseases/conditions (valvular heart disease, coronary heart disease, arrhythmias, ischemia, cardiomyopathies and heart failure and cardiac surgeries); b) hematological diseases/conditions (platelet disorders, sickle cell anemia or thalassemia); c) Down syndrome, according to patient report or research in medical records; d) liver disease (cirrhosis); e) asthma, according to patient report or research in medical records; f) type 1, 2 or gestational diabetes; g) neurological disease (motor deficit, epilepsy, myasthenia gravis, cerebrovascular accident or cases of toxoplasmosis); h) respiratory diseases (pneumonia, bronchitis or chronic obstructive pulmonary disease); i) immunosuppression (lupus, rheumatoid arthritis, Crohn’s disease, hemolytic anemia, spondyloarthritis, psoriatic arthritis, systemic sclerosis, Sjögren’s syndrome, inflammatory myopathies, and vasculitis); j) kidney disease; l) obesity (body mass index greater than or equal to 30 kg/m^2^); m) hypertension, as reported by the patient or researched in medical records; n) HIV/AIDS, as reported by the patient or researched in medical records; o) drug/substance abuse (reported sustained consumption of alcohol, tobacco, and/or illicit drugs, such as marijuana, cocaine, crack, etc.); p) psychiatric disorder (mood disorders and/or thought disorders, as reported by the patient); q) thyroid disease (hypothyroidism or hyperthyroidism), and; r) pregnancy-specific hypertensive disease. These variables were considered in the prediction study.

The following were considered as outcomes: a) The causative agent of SARS (influenza or COVID-19); b) The outcome of the SARS case during pregnancy (cure, death and/or death from another cause).

### Data Analysis and Processing

For the initial study of sociodemographic and epidemiological variables, we calculated the absolute frequency and percentage, considering their characteristics of qualitative variables (nominal or ordinal). We performed the same action for all predictive variables under investigation. Next, we studied the association of predictive variables (the presence of comorbidities was dichotomized as yes or no) with the causative agent of SARS (dichotomized as influenza or COVID-19) using contingency tables and the chi-square test. The mortality rate was calculated according to the following formula:


$$\frac{\begin{array}{c} N^{\circ }\;deaths\;of\;pregnant\;women\;with\;specific\\ comorbidities\;in\;the\;period \end{array}}{\begin{array}{c} N^{\circ }\;deaths\;of\;pregnant\;women\;with\;some\\ comorbidity\;in\;the\;period \end{array}}$$

The data used in the calculation refer to January 2020-March 2022. The correlation between the number of days of hospital epidemiological investigation and the presence of comorbidities/health factors was examined using Spearman’s correlation coefficient and the nonparametric Kruskal-Wallis test, correlating the median length of hospital stay (in days) with the presence of health factors or comorbidities. We used the Jamovi version 1.6.23 and JASP 1.0 (free versions) in the analyzes, always considering a 95% confidence interval in the measurements.

### Ethical Aspects

Approval from a Research Ethics Committee was not required for the development of this study because open access data from the website of the Brazilian Ministry of Health (https://opendatasus.saude.gov.br/dataset/srag-2021-a-2023) were used.

## RESULTS

### Characteristics of Pregnant Women

This study included 1,152 pregnant women. Most were in the second trimester of pregnancy (67.2%), had brown skin color (55.7%), an average of 12 years of schooling (56.8%), and age between 30 and 59 years (mean of 29.8 ± 7.1). Almost all lived in urban areas (92.7%), and did not work or have any direct contact with animals (99.2%). The cases studied were not nosocomial SARS (98.4%) nor part of a specific outbreak (80.0%) (Table 1).

**Table 1:** Sociodemographic and epidemiological characteristics of pregnant women – Brasília, DF, Brazil, 2023. (n = 1,152).

Variable	Frequency	Percentage
**Gestational age**		
First trimester	102	8.9
Second trimester	275	23.9
Third trimester	775	67.2
**Age group**		
≤ 14 years	03	0.3
15-19 years	92	8.0
20-29 years	446	38.7
30-59 years	611	53.0
**Skin color/race**		
White	403	35.0
Black	90	7.8
Yellow	08	0.7
Brown	642	55.7
Indigenous	09	0.8
**Educational level**		
Illiterate	05	0.4
Up to 5 years of schooling	84	7.3
Up to 9 years of schooling	225	19.5
Up to 12 years of schooling	654	56.8
Higher education	184	16.0
**Residence area**		
Urban	1,068	92.7
Rural	73	6.3
Peri-urban	11	1.0
**Region of the country**		
North	152	13.2
Northeast	304	26.4
Central-West	169	14.7
Southeast	398	34.5
South	129	11.2
**COVID-19 outbreak**		
Yes	230	20.0
No	922	80.0
**Hospital-acquired SARS**		
Yes	18	1.6
No	1,134	98.4
**Direct contact with poultry, swine or other animals**		
Yes	10	0.8
No	1,142	99.2

Of the total, 97% of the sample had a confirmed diagnosis of COVID-19. Clinical variables indicated that the average body mass index (BMI) of women was 32.1 kg/m^2^ (SD ± 13.7). Approximately 60% of the medical records contained information on immunization against COVID-19, of which 24.3% indicated a complete vaccination schedule. Most pregnant women had no diagnosis of chronic noncommunicable diseases (53.3%). A smaller proportion had one (35.7%), two (9.4%), three (1%), four (0.2%) or five (0.1%) chronic conditions. The most common comorbidities were diabetes (26.8%), cardiovascular disease (17.1%), obesity (12.6%), arterial hypertension (7.5%) and asthma (10.4%). Overall, 28.6% of the sample had high-risk pregnancies (Table 2).

Regarding those infected with the influenza virus, the sample size was smaller (n = 34).

Regarding the etiological agent SARS (influenza or COVID-19), we identified significant statistical associations with the following comorbidities: diabetes (p = 0.023), neurological disease (p = 0.001) and substance use (p = 0.001) (Table 2).

In the sample analyzed, we did not identify cases of pregnant women with influenza-related SARS and the comorbidities: Down syndrome, liver disease, kidney disease, HIV/AIDS and mental disorder.

**Table 2:** Association between chronic health conditions/comorbidities and SARS in pregnant women – Brasília, DF, Brazil, 2023. (n = 1.152).

Comorbidities	Influenza** n (%)	COVID-19** n (%)	p-value^†^
Cardiovascular disease	3 (0.2)	194 (16.9)	0.222
Hematological disease	1 (0.0)	14 (1.2)	0.740
Asthma	6 (0.5)	114 (9.9)	0.159
Diabetes	4 (0.3)	303 (26.5)	**0.023**
Neurological disease	4 (0.3)	18 (1.6)	**0.001**
Respiratory disease	2 (0.1)	18 (1.6)	0.281
Immunosuppression	2 (0.1)	32 (2.9)	0.701
Obesity	3 (0.2)	142 (12.4)	0.433
High blood pressure	2 (0.1)	84 (7.4)	0.569
Gestational diabetes	1 (0.0)	47 (4.1)	0.607
Substance use and abuse*	3 (0.2)	13 (1.3)	**0.001**
Thyroid disease	2 (0.1)	44 (3.9)	0.721
Pregnancy-specific hypertensive disease	1 (0.0)	49 (4.2)	0.576

*Cases of smoking, alcoholism or use of illicit drugs,

^†^Chi-square test.

**The tables were prepared based on simultaneous affirmative responses, both for the presence of comorbidities and for the identification of the causative agent of SARS.

### Characteristics of Sars

The main complaints of hospitalized pregnant women were cough (69.3%), fever (60.2%), dyspnea (53.1%), difficulty breathing (44.7%), pharyngitis (23.4%), vomiting (13%), loss of smell (12.8%), loss of taste (12%), diarrhea (11.5%), fatigue (10%) and abdominal pain (5.1%). Although many women had adequate oxygen levels (70.3%), most were still hospitalized (97.4%) and some of them were admitted to intensive care units (21.3%).

Regarding oxygen therapy, more than half of patients did not require invasive respiratory support (55.4%). Of those who did, 28.3% used non-invasive support and another 8.6% required intubation. About a quarter of the patients received antiviral treatment for influenza (23.4%); oseltamivir was the most commonly used drug (90.6%).

In terms of diagnostic measures, chest X-rays were not performed in almost half of the cases (48.7%). However, a biological sample was collected in 95.4% of the sample, usually from the nose and throat (75.2%). The main diagnostic tool were laboratory tests (95.1%). The results of the RT-PCR test showed that 72.1% of the patients had a positive RT-PCR COVID-19 test, among which 97.0% (n = 1,118) had SARS caused by COVID-19. A small percentage had influenza (3.0%, n = 34). The mortality rate for SARS was 7% (n = 81), and among these cases, 97% (n = 78) died of COVID-19.

### Epidemiological Surveillance

The mean time for diagnostic confirmation of SARS cases in pregnant women was 42.6 days (SD ± 76.2). We also identified a weak but statistically significant incremental correlation between the number of days of epidemiological investigation and the number of comorbidities (Spearman’s rho = 0.055, p = 0.030). In patients with heart disease, the epidemiological investigation lasted up to 24 days (median), while in pregnant women with preserved cardiovascular health (*χ* ^2^ = 8.48, df = 2, p = 0.014) it took up to 20 days.

The completion of the epidemiological study of SARS took longer for Black women (23 days) than for women of other ethnicities: Brown women (21 days), Indigenous women (21 days), White women (19 days) and Yellow women (18 days) (*χ* ^2^ = 14.7, df = 5, p = 0.012). The investigation also took longer in hospitals located in the North and South regions of Brazil (23 days) compared to hospitals in other regions, where it lasted 16 days (*χ* ^2^ = 12.9, df = 4, p = 0.011).

**Figure 1:**
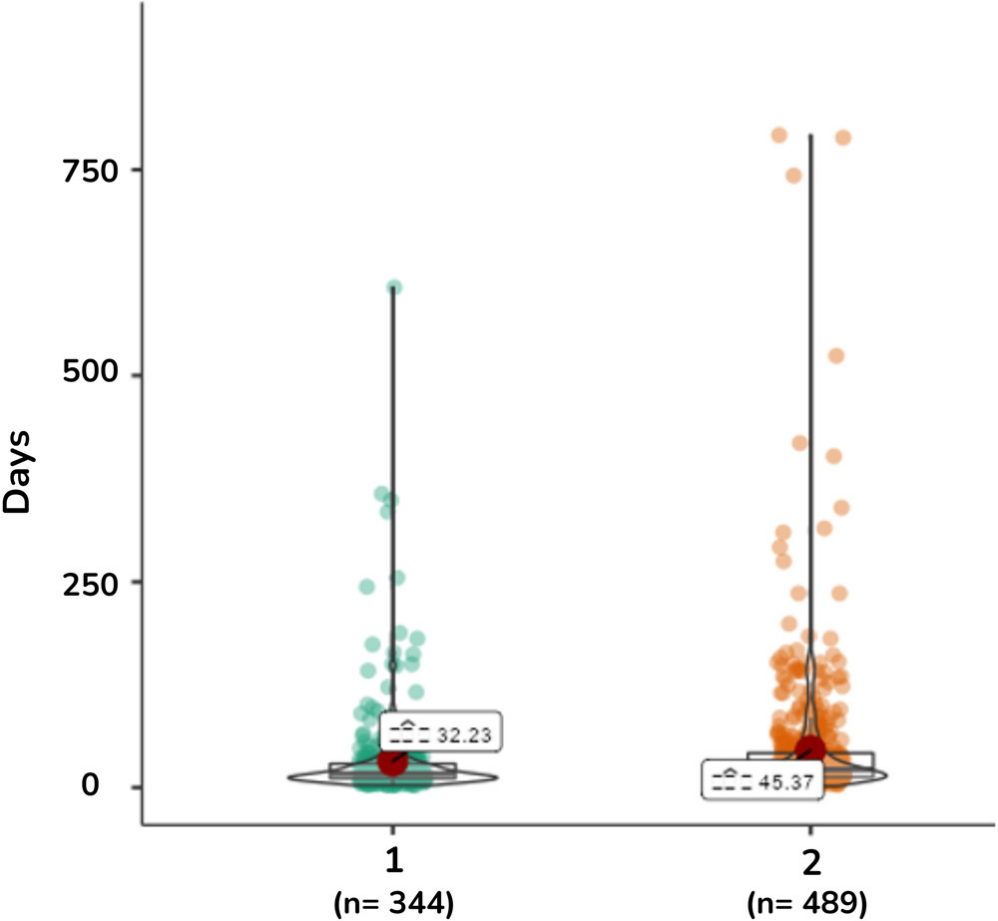
Length of hospital stay due to SARS in pregnant women according to COVID-19 vaccination status. Brazil, 2023.

We also found that the epidemiological surveillance time for cases was longer in pregnant women with three comorbidities (39 days) compared to those with no comorbidities (20 days), one (20 days) or two (24 days) comorbidities (*χ* ^2^ = 12.6, df = 4, p = 0.008). The epidemiological investigation period for SARS was longer in pregnant women who were not vaccinated against COVID-19 (22 days), compared to those who were vaccinated (17 days) (U-Whitney = 2.600, df = 837, p < 0.001) (Figure 1).

According to our analysis, the predictor variable high-risk pregnancy (yes/no) did not show a statistically significant association with either the causative agent of SARS (*χ* ^2^ = 2.045, df = 2, p = 0.360) or the outcome of the case of gestational SARS (cure/death) (*χ* ^2^ = 2.543, df = 1, p = 0.111).

## DISCUSSION

In the sample we analyzed, the cases of pregnant women infected with influenza (n = 34) were close to what is recommended by the central limit theory: observations with a quantity <30 increase statistical uncertainty. That is, making arguments based on such a small sample could lead us to commit a type II error in biostatistics. Thus, we focus our argument on the association of comorbidities with cases of SARS related to COVID-19.

The pregnant women with SARS were mostly Brown, living in urban areas, young adults, and in the second trimester of pregnancy. The diagnosis of COVID-19 was identified in 97% of the sample, and the pregnancies of almost 1/3 of women were high-risk. The course of SARS-CoV-2 infection appears to be more severe in pregnant women, and this condition can generate a greater risk of spontaneous abortion, premature birth, and increased length of hospital stay. This increases healthcare costs and demands monitoring, treatment, and rearrangements in the organization of specialized care for the mother-baby dyad^(14)^. The situation is also challenging, as it increases the chances of anxiety and depression among women^(15,16)^.

This population is more susceptible to respiratory infections due to the physiological, anatomical, and immunological changes that occur during pregnancy and childbirth^(17)^. This vulnerability is aggravated by the presence of pre-existing chronic conditions that exacerbate inflammatory cascades, leading to negative clinical implications for both the mother and the fetus. Pregnant women are also more likely to develop cytokine storms indicative of severe viral infection, which can induce vasoconstriction and intrauterine fetal growth restriction^(18)^.

Diabetes, cardiovascular disease and obesity were the more common pre-existing chronic conditions among the pregnant women in this study. This may be related to the change in daily life activities due to the lockdowns established by public agencies, the interruption of routine prenatal care and management of health conditions in health services, and the increase in sedentary lifestyle and excessive weight gain in the early years of the COVID-19 pandemic. In addition, gestational diabetes rates also increased between 2020 and 2021 in different countries around the world^(19)^.

In pregnant women, both obesity and diabetes increase pro-inflammatory cytokines and adipokines, such as TNF-*α*, IL-6, IL-1*β*, leptin, and resistin, which are involved in the inflammatory response. Furthermore, insulin resistance generated by the two aforementioned diseases can affect the functioning of immune system cells and contribute to reducing the effectiveness of vaccines in this population^(20)^. This may have compromised the survival rate in the sample studied, given the low rate of women with a complete vaccination schedule.

In addition to the association found between COVID-19 and diabetes (p = 0.023), the association between substance use and abuse (p = 0.001) is also noteworthy. A Canadian study showed that stronger symptoms of depression and financial difficulties were associated with increased substance use and abuse during pregnancy^(21)^. In turn, another North American study showed that mental/emotional health problems prior to the COVID-19 pandemic were significantly associated with greater use and abuse of substances to deal with stress and depressive symptoms caused by the pandemic. This maximizes changes in the central nervous system and can generate risks such as dependence on substances that are harmful to health. In high concentrations, the use of substances such as cocaine and its derivatives during pregnancy is linked to high concentrations of estrogen, which modify the functioning of the respiratory system, leading to increased local vascularization and greater absorption of these drugs. In the use and abuse of tobacco, the accumulation of nicotine can cross the placental barrier and compromise fetal maturation^(22)^. In this sense, discussions and interventions addressing the prevention and control of stress, anxiety, depression and risk behaviors must be addressed with this population.

Associations between the diagnosis of COVID-19 and neurological diseases (p = 0.001) were present among pregnant women. Evidence shows that ischemic stroke is the most common neurological complication of COVID-19, affecting up to 6% of all patients with the infection. In pregnant women specifically, a scoping review identified records of alterations such as delirium, encephalopathy, cerebrovascular disease and Guillain-Barré syndrome associated with COVID-19^(23)^. On the other hand, a study conducted in northeastern Brazil found no difference in the manifestation of neurological complications when comparing pregnant and non-pregnant women with COVID-19^(24)^. However, data on the subject are still scarce and require further investigation.

The most common clinical complaints presented by pregnant women were similar to those found in other populations; cough, fever, and dyspnea were the most prevalent symptoms. A cross-sectional study in Iran also showed that cough and dyspnea were the most common symptoms among women with the SARS-CoV-2 virus, increasing the demand for oxygen therapy^(25)^. In this study, ventilatory support was required by 28.3% of pregnant women, and almost 10% of them required invasive ventilation. Data from a study conducted in Asia showed that the rate of low-flow oxygen support was 52.7% among hospitalized pregnant women, and that 2.7% of the population required intubation^(25)^.

In most cases, the administration of oxygen therapy and the management of severe respiratory changes are performed in intensive care units. In this study, 21.3% of women were hospitalized in these units. These results were lower than those of an Iranian study, in which the hospitalization rate was 36%^(25)^. A systematic review that investigated maternal and neonatal complications due to COVID-19 found that the admission of pregnant women to intensive care units was common in most studies on the subject, with an occurrence rate of 52.9%. Furthermore, other negative outcomes identified were premature birth, maternal death, premature rupture of membranes, preeclampsia, intrauterine growth restriction, and stillbirth^(26)^. This demonstrates the importance of systematized prenatal care under the watchful eye of professionals who use advanced practices in their healthcare, such as nurses.

In this study, maternal mortality was 7%. A Brazilian study that analyzed the first two years of the COVID-19 pandemic showed that the mortality rate increased twice (7.7 to 15.4%) between the first (2020) and second (2021) waves of cases of the disease, and the rates were even higher in women with comorbidities^(27)^. Similar data were also found in India^(28)^. The time taken to diagnose the disease is also noteworthy. According to the analysis, it took 42 days for the case to be confirmed. This was influenced by the existence of comorbidities, the skin color of the pregnant woman, and the region of the country. The delay in epidemiological conclusion in women with multiple health conditions may be related to the greater complexity in conducting care and in the differential diagnoses listed. Regarding the skin color of pregnant women, results from another Brazilian study show that racism can be the major trigger for negative outcomes. The mortality rate for COVID-19 in White women was 14% lower compared to Black and Brown women. In the puerperal period, the probability of death for Black women was 62% higher^(29)^.

These conditions highlight the need to identify social determinants of health in the development and implementation of care aimed at promoting health, preventing diseases and conditions, and managing clinical changes and associated chronic illnesses, especially in populations at-risk, such as pregnant women. In view of this, the leading role of nursing professionals in primary healthcare and in the hospital context with critical care aimed at the mother-baby dyad, is highlighted. Furthermore, the inclusion of nurses in epidemiological surveillance sites should be encouraged, as well as a greater in-depth study of the diligence in the processes of identification, notification, and dissemination of data.

Although our study provided a better understanding of the association between chronic health conditions and the diagnosis of SARS in pregnant women, a cautious interpretation of the findings is required, and some limitations need to be highlighted. In this study, it was not possible to collect specific clinical and laboratory indicators regarding the health conditions presented. Information on substance use and abuse was based on the pregnant women’s self-report, which may have led to potential omissions in the responses. Identifying the reasons for the delay in the diagnosis of SARS cases in pregnant women and the influencing factors were also some of the limitations found. Despite these deficiencies, the results presented are valuable for guiding nursing care, and that of other areas, and significant for the development of public health policies aimed at the population of interest.

## CONCLUSION

The previous presence of diabetes mellitus, neurological disease, and substance use and abuse was involved in the diagnosis, but not in the death of pregnant women with SARS. Further research using population-based data needs to be designed to clarify the factors influencing the processes of disease and mortality among pregnant and puerperal women.
