# Supplementary material for: Association between chronic health conditions and severe acute respiratory syndrome in pregnant women: an exploratory study
Source: Rev Esc Enferm USP. 2024 Nov 22;58:e20230336. doi: 10.1590/1980-220X-REEUSP-2023-0336en (PMC11583968; doi:10.1590/1980-220X-REEUSP-2023-0336en)
Supplement: Translation

# Translation: Associação entre condições crônicas de saúde e a síndrome respiratória aguda grave em gestantes: estudo exploratório

**DOI:** 10.1590/1980-220X-REEUSP-2023-0336pt

**Authors:** José Cláudio Garcia Lira, Márcio Flávio Moura de Araújo, Flavia Paula Magalhães Monteiro, Roberto Wagner Júnior Freire de Freitas, Carla Regina de Sousa Teixeira, Floriacy Stabnow Santos, Ana Cristina Pereira de Jesus Costa, Marcelino Santos, Lívia Maia Pascoal

**Affiliations:** 1 Universidade Federal do Piauí, Departamento de Enfermagem, Floriano, PI, Brasil.; 2 Fundação Oswaldo Cruz, Eusébio, CE, Brasil.; 3 Universidade da Integração Internacional da Lusofonia Afro-Brasileira, Redenção, CE, Brasil.; 4 Universidade de São Paulo, Departamento de Enfermagem, Ribeirão Preto, SP, Brasil.; 5 Universidade Federal do Maranhão, Imperatriz, MA, Brasil.

**Keywords:** COVID-19, Síndrome Respiratória Aguda Grave, Diabetes Mellitus, Gravidez

## Abstract

**Objetivo:**

Analisar a associação entre condições crônicas de saúde e a síndrome respiratória aguda grave em gestantes.

**Método:**

Estudo retrospectivo, exploratório realizado com 1.152 gestantes de todos os 27 estados do Brasil que procuraram tratamento hospitalar e foram diagnosticadas com síndrome respiratória aguda grave entre 2020 e 2022. Foram utilizados dados públicos do Sistema de Informação de Vigilância Epidemiológica da Gripe (SIVEP-Gripe) do Ministério da Saúde do Brasil. Realizamos testes não paramétricos para a interpretação dos dados.

**Resultados:**

A taxa de mortalidade de gestantes por síndrome respiratória aguda grave no Brasil foi de 7%. A síndrome respiratória aguda grave estava estatisticamente associada à presença prévia de diabetes (p = 0,023), doença neurológica (p = 0,001) e uso de drogas (p = 0,001). A investigação epidemiológica dos casos de síndrome respiratória demorou mais tempo em gestantes negras (p = 0,012), não vacinadas (p < 0,001) e residentes na região norte e sul do país (p = 0,011).

**Conclusão:**

A síndrome respiratória aguda grave foi mais comum em gestantes com diabetes, doença neurológica e que faziam uso de drogas. Todavia, essas condições não levaram ao aumento no número de mortes.

## INTRODUÇÃO

As gestantes estão entre os grupos mais vulneráveis ao acometimento de Síndromes Respiratórias Agudas Grave (SRAG), quando comparadas com a população em geral, independentemente do agente etiológico^(1,2)^. A procura por cuidados pré-natais intensifica-se na presença do vírus SARS-CoV-2, causador da COVID-19, levando ao aumento na complexidade terapêutica e exigindo intervenções ainda mais prementes^(3,4)^.

No Brasil, a taxa de mortalidade materna por COVID-19 teve um salto nos meses iniciais após o aparecimento dos primeiros casos da doença no país, e foi duas vezes maior no ano seguinte^(5)^. Ademais, evidências têm mostrado que o número de hospitalizações, necessidades de cuidados críticos, do uso de substâncias vasoativas e de ventilação mecânica relacionados à essa infecção parecem ser mais elevados entre mulheres grávidas. Dentre as consequências associadas, pontua-se um maior índice de trabalho de parto prematuro, pré-eclâmpsia e admissão neonatal em unidades de terapia intensiva^(6, 7, 8)^.

Os efeitos desfavoráveis estão estreitamente relacionados a comportamentos de risco, como o uso e abuso de substâncias, o excesso de peso e a condições crônicas de saúde, como o diabetes^(6)^. Além disso, o período gravídico-puerperal tem se tornado centro de discussões na assistência à saúde por induzir alterações fisiológicas e respostas que aumentam o risco de complicações respiratórias, cardiovasculares, neurológicas e metabólicas em especial, pelos potenciais desfechos envolvidos. Mesmo assim, os dados disponíveis sobre as características específicas de gestantes que deveriam receber maior atenção dos profissionais de saúde em hospitais e maternidades em todo o mundo são limitados^(9, 10, 11, 12, 13)^.

Dessa forma, faz-se imperioso que tais informações sejam exploradas a fim de se obter uma compreensão mais profunda sobre a ocorrência de SRAG na gravidez e condições relacionadas para o desenvolvimento de cuidados em saúde. O objetivo desse estudo foi analisar a associação entre condições crônicas de saúde e a SRAG em gestantes.

## MÉTODO

### Desenho de Estudo

Trata-se de um estudo retrospectivo, multicêntrico e documental, baseado na análise dos dados durante o período de 2020 a 2022 do Sistema de Informação para Vigilância Epidemiológica da Gripe (SIVEP-Gripe), do Ministério da Saúde do Brasil.

### Local, População e Critérios de Seleção

Foram incluídas todas as gestantes com SRAG decorrente de infecção por influenza ou COVID-19 que procuraram atendimento hospitalar entre os anos de 2020 e 2022 nos 27 estados brasileiros e no Distrito Federal. Para serem incluídas na pesquisa, as gestantes deveriam ter uma ficha de investigação epidemiológica para SRAG em ambiente hospitalar disponível. Para fins epidemiológicos, foram considerados os casos de SRAG em gestantes internadas com febre, mesmo que relatada, acompanhada de tosse ou dor de garganta e que apresentassem dispneia ou saturação de oxigênio (O_2_) < 95%; ou dificuldade respiratória; ou que evoluíram para óbito por SRAG, independentemente da internação.

### Definição da Amostra

A amostra foi composta por 1.152 gestantes que haviam sido hospitalizadas com diagnóstico de SRAG. A inclusão das participantes se deu a partir da análise de todas as fichas completas notificadas no sistema SIVEP-Gripe, no referido recorte temporal.

### Coleta de Dados

Os dados foram coletados no sistema SIVEP-Gripe em janeiro de 2023, com apoio de um formulário contendo 41 questões aplicado previamente por profissionais de saúde da vigilância epidemiológica de distintos serviços de saúde (unidade básica de saúde, ambulatórios, hospitais e unidades de reabilitação). Questões sociodemográficas contendo informações acerca da idade, faixa etária, idade gestacional, cor da pele, escolaridade, região e notificação de infecção hospitalar foram consideradas, bem como questões relacionadas a comorbidades ou fatores de saúde pré-existentes, a saber: a) doenças/condições cardiovasculares (valvopatias, doenças coronarianas, arritmias, isquemias, cardiomiopatias e insuficiência cardíaca e cirurgias cardíacas); b) doenças/condições hematológicas (distúrbios plaquetários, anemia falciforme ou talassemia); c) Síndrome de Down, conforme relato da paciente ou pesquisa em prontuário; d) doença hepática (cirrose); e) asma, conforme relato da paciente ou pesquisa em prontuário; f) Diabetes tipo 1, 2 ou gestacional; g) doença neurológica (déficit motor, epilepsia, miastenia gravis, acidente vascular cerebral ou casos de toxoplasmose); h) doenças respiratórias (pneumonia, bronquite ou doença pulmonar obstrutiva crônica); i) imunossupressão (lúpus, artrite reumatoide, doença de Crohn, anemia hemolítica, espondilartrite, artrite psoriática, esclerose sistêmica, síndrome de Sjögren, miopatias inflamatórias e vasculite); j) doença renal; l) obesidade (índice de massa corporal maior ou igual a 30 Kg/m^2^); m) hipertensão, conforme relato da paciente ou pesquisa em prontuário; n) HIV/AIDS, conforme relato da paciente ou pesquisa em prontuário; o) uso abusivo de drogas/substâncias (relato de consumo sustentado de álcool, tabaco e/ou drogas ilícitas, como maconha, cocaína, crack, etc.); p) transtorno psiquiátrico (transtornos de humor e/ou transtornos de pensamento, conforme relato da paciente); q) doença da tireoide (hipotireoidismo ou hipertireoidismo), e; r) doença hipertensiva específica da gestação. Essas variáveis foram consideradas no estudo de predição.

Como desfechos, considerou-se: a) Agente causador da SRAG (influenza ou COVID-19); b) Evolução do caso de SRAG gestacional (cura, óbito e/ou óbito por outra causa).

### Análise e Tratamento dos Dados

Para o estudo inicial das variáveis sociodemográficas e de caráter epidemiológico, calculamos a frequência absoluta e a percentual, considerando suas características de variável qualitativa (nominal ou ordinal). Executamos essa mesma ação para todas as variáveis preditivas sob investigação. Na sequência, realizamos o estudo da associação das variáveis preditoras (a presença de comorbidades foi dicotomizada em sim ou não) com o agente causador da SRAG (dicotomizada em influenza ou COVID-19) por meio de tabelas de contingência e teste de qui-quadrado. A taxa de mortalidade foi calculada conforme a seguinte fórmula:


$$\frac{\begin{array}{c} N^{\circ }\;óbitos\;de\;fes\;ten\;tes\;com\;comorbidade\\ específica\;no\;período \end{array}}{\begin{array}{c} N^{\circ }\;óbitos\;gestantes\;com\;alguma\\ comorbidade\;no\;período \end{array}}$$

Os dados empregados no cálculo são referentes a janeiro de 2020 até março de 2022. Também foi examinada a correlação entre o número de dias de investigação epidemiológica hospitalar e a presença de comorbidades/fatores de saúde por meio do coeficiente de correlação de Spearman e do teste não paramétrico de Kruskal-Wallis, correlacionando a mediana do tempo de internação (em dias) com a presença de fatores de saúde ou comorbidades. Utilizamos as versões gratuitas Jamovi 1.6.23 e JASP 1.0 para as análises, considerando sempre um intervalo de confiança de 95% nas mensurações.

### Aspectos Éticos

Esta pesquisa não necessitou de aprovação de um Comitê de Ética em Pesquisa por se tratar de um estudo com dados de acesso aberto do site do Ministério da Saúde do Brasil (https://opendatasus.saude.gov.br/dataset/srag-2021-a-2023).

## RESULTADOS

### Características das Gestantes

Nessa pesquisa, foram incluídas 1.152 gestantes. A maioria estava no segundo trimestre de gestação (67,2%), cor de pele parda (55,7%), escolaridade média de 12 anos (56,8%) e idade entre 30 e 59 anos (média de 29,8 ± 7,1). Quase a totalidade das investigadas residia em áreas urbanas (92,7%) e não trabalhavam ou tinham qualquer contato direto com animais (99,2%). Os casos estudados não eram SRAG nosocomial (98,4%), nem parte de um surto específico (80,0%) (Tabela 1).

**Tabela 1:** Características sociodemográficas e epidemiológicas das gestantes – Brasília, DF, Brasil, 2023. (n = 1.152).

Variável	Frequência	Porcentagem
**Idade gestacional**		
Primeiro trimestre	102	8,9
Segundo trimestre	275	23,9
Terceiro trimestre	775	67,2
**Faixa etária**		
≤ 14 anos	03	0,3
15-19 anos	92	8,0
20-29 anos	446	38,7
30-59 anos	611	53,0
**Cor da pele/raça**		
Branca	403	35,0
Preta	90	7,8
Amarela	08	0,7
Parda	642	55,7
Indígena	09	0,8
**Nível educacional**		
Analfabeto	05	0,4
Até 5 anos de estudo	84	7,3
Até 9 anos de estudo	225	19,5
Até 12 anos de estudo	654	56,8
Educação superior	184	16,0
**Zona de residência**		
Urbana	1.068	92,7
Rural	73	6,3
Periurbana	11	1,0
**Região do país**		
Norte	152	13,2
Nordeste	304	26,4
Centro-oeste	169	14,7
Sudeste	398	34,5
Sul	129	11,2
**Surto de COVID-19**		
Sim	230	20,0
Não	922	80,0
**SARS adquirida em hospital**		
Sim	18	1,6
Não	1.134	98,4
**Contato direto com aves, suínos ou outros animais**		
Sim	10	0,8
Não	1.142	99,2

Do total, 97% da amostra teve diagnóstico confirmado de COVID-19. As variáveis clínicas indicaram que índice de massa corpórea (IMC) médio das mulheres foi de 32,1kg/m^2^ (DP ± 13,7). Cerca de 60% dos prontuários continham informações sobre imunização contra a COVID-19, dos quais 24,3% indicavam esquema vacinal completo. A maioria das gestantes não apresentava diagnóstico de doenças crônicas não transmissíveis (53,3%). Uma proporção menor tinha uma (35,7%), duas (9,4%), três (1%), quatro (0,2%) ou cinco (0,1%) condições crônicas. As comorbidades mais comuns foram diabetes (26,8%), doenças cardiovasculares (17,1%), obesidade (12,6%), hipertensão arterial (7,5%) e asma (10,4%). No geral, 28,6% da amostra teve gravidez de alto risco (Tabela 2).

Quanto àquelas infectadas com o vírus influenza, o quantitativo amostral foi menor (n = 34).

Quanto ao agente etiológico SRAG (influenza ou COVID-19), identificamos associações estatísticas significativas com as seguintes comorbidades: diabetes (p = 0,023), doença neurológica (p = 0,001) e uso de substâncias (p = 0,001) (Tabela 2).

Na amostra analisada não identificamos casos de gestantes com SRAG relacionados ao vírus influenza com as comorbidades Síndrome de Down, Doença hepática, Doença renal, HIV/AIDS e Distúrbio mental.

**Tabela 2:** Associação entre condições crônicas de saúde/comorbidades e SRAG em gestantes – Brasília, DF, Brasil, 2023. (n = 1.152).

Comorbidades	Influenza** n (%)	COVID-19** n (%)	p-valor^†^
Doença cardiovascular	3 (0,2)	194 (16,9)	0,222
Doença hematológica	1 (0,0)	14 (1,2)	0,740
Asma	6 (0,5)	114 (9,9)	0,159
Diabetes	4 (0,3)	303 (26,5)	**0,023**
Doença neurológica	4 (0,3)	18 (1,6)	**0,001**
Doença respiratória	2 (0,1)	18 (1,6)	0,281
Imunossupressão	2 (0,1)	32 (2,9)	0,701
Obesidade	3 (0,2)	142 (12,4)	0,433
Hipertensão arterial	2 (0,1)	84 (7,4)	0,569
Diabetes gestacional	1 (0,0)	47 (4,1)	0,607
Uso e abuso de substâncias*	3 (0,2)	13 (1,3)	**0,001**
Doença da tireoide	2 (0,1)	44 (3,9)	0,721
Doença hipertensiva específica da gravidez	1 (0,0)	49 (4,2)	0,576

*Casos de tabagismo, alcoolismo ou uso de drogas ilícitas,

^†^Teste qui-quadrado.

**A composição das tabelas foi baseada nas respostas afirmativas simultâneas, tanto para a presença de comorbidades, quanto para a identificação do agente causador de SRAG.

### Características da Srag

As principais queixas das gestantes hospitalizadas foram tosse (69,3%), febre (60,2%), dispneia (53,1%), dificuldade respiratória (44,7%), faringite (23,4%), vômito (13%), perda de olfato (12,8). %), perda de paladar (12%), diarreia (11,5%), fadiga (10%) e dor abdominal (5,1%). Apesar de muitas mulheres apresentarem níveis adequados de oxigênio (70,3%), a maioria ainda estava internada (97,4%) e parte delas internada em unidades de terapia intensiva (21,3%).

Em relação à oxigenoterapia, mais da metade das pacientes não necessitaram de suporte respiratório invasivo (55,4%). Das que o fizeram, 28,3% utilizaram suporte não invasivo e outras 8,6% necessitaram de intubação. Cerca de um quarto das pacientes recebeu tratamento antiviral para gripe (23,4%), sendo o Oseltamivir o medicamento mais utilizado (90,6%).

Em termos de medidas diagnósticas, a radiografia de tórax não foi realizada em quase metade dos casos (48,7%). Contudo, uma amostra biológica foi coletada em 95,4% da amostra, geralmente do nariz e da garganta (75,2%). A principal ferramenta diagnóstica foram os exames laboratoriais (95,1%). Os resultados do teste RT-PCR mostraram que 72,1% das pacientes apresentaram teste RT-PCR COVID-19 positivo, entre os quais 97,0% (n = 1.118) tiveram SRAG causada por COVID-19. Uma pequena percentagem teve influenza (3,0%, n = 34). A taxa de mortalidade por SRAG foi de 7% (n = 81), e a morte de 97% (n = 78) destes casos foi por COVID-19.

### Vigilância Epidemiológica

A média de confirmação diagnóstica dos casos de SRAG em gestantes foi 42,6 dias (DP ± 76,2). Identificamos também uma correlação incremental fraca, mas estatisticamente significativa, entre o número de dias de investigação epidemiológica e o número de comorbidades (rho de Spearman = 0,055, p = 0,030). Nas pacientes cardiopatas, a investigação epidemiológica durou até 24 dias (mediana), enquanto nas gestantes com saúde cardiovascular preservada (χ^2^ = 8,48, gl = 2, p = 0,014), a conclusão da investigação epidemiológica demorou até 20 dias.

O estudo epidemiológico da SRAG demorou mais para ser concluído para mulheres negras (23 dias), do que para mulheres de outras etnias: mulheres pardas (21 dias), indígenas (21 dias), brancas (19 dias) e amarelas (18 dias) (χ^2^ = 14,7, gl = 5, p = 0,012). A investigação também demorou mais nos hospitais localizados nas regiões Norte e Sul do Brasil (23 dias) em comparação aos hospitais de outras regiões, onde durou 16 dias (χ^2^ = 12,9, gl = 4, p = 0,011).

Verificamos também que o tempo de vigilância epidemiológica dos casos foi maior nas gestantes com três comorbidades (39 dias) em comparação com aquelas sem nenhuma comorbidade (20 dias), uma (20 dias) ou duas (24 dias) comorbidades (χ^2^ = 12,6, df = 4, p = 0,008). O período de investigação epidemiológica para SRAG foi maior nas gestantes que não foram vacinadas para COVID-19 (22 dias), em comparação às que foram vacinadas (17 dias) (U-Whitney = 2.600, gl = 837, p < 0,001) (Figura 1).

**Figura 1:** Período de internação por SRAG em gestantes segundo situação vacinal contra COVID-19. Brasil, 2023.

Conforme nossa análise, a variável preditora gravidez de alto risco (sim/não) não apresentou associação estatisticamente significativa nem com o agente causador da SRAG (χ^2^ = 2,045, gl = 2, p = 0,360), nem com a evolução do caso de SRAG gestacional (cura /óbito) (χ^2^ = 2,543, gl = 1, p = 0,111).

## DISCUSSÃO

Na amostra que analisamos, os casos de gestantes infectadas com influenza (n = 34) ficaram próximo ao preconizado pela teoria central do limite: observações com quantitativo < 30 elevam a incerteza estatística. Isto é, tecer argumentações partindo de amostra tão pequena poderia nos influenciar a cometer erro do tipo II em bioestatística. Dessa maneira, centramos nossa argumentação na associação das comorbidades com os casos de SARG relacionados a COVID-19.

As gestantes com a SRAG eram, em sua maioria, de cor de pele parda, residentes em áreas urbanas, adultas jovens, e estavam no segundo trimestre de gestação. O diagnóstico de COVID-19 foi identificado em 97% da amostra, e a gravidez de quase 1/3 das mulheres era de alto risco. O curso da infecção por SARS-CoV-2 em gestantes parece se apresentar de forma mais grave, e essa condição pode gerar um maior risco de abortamento espontâneo, parto prematuro e aumento no tempo de hospitalização. Isso eleva os custos com cuidados em saúde e demanda intervenções de acompanhamento, tratamento e rearranjos na organização de assistência especializada destinada ao binômio mãe-bebê^(14)^. A situação também é desafiadora, pois agrava as chances de aparecimento da ansiedade e depressão entre as mulheres^(15,16)^.

Essa população está mais suscetível a infecções respiratórias pela ocorrência de alterações fisiológicas, anatômicas e imunológicas durante a gravidez e o parto^(17)^. A vulnerabilidade é agravada pela presença de condições crônicas pré-existentes que exacerbam cascatas inflamatórias, levando a implicações clínicas negativas tanto para a mãe quanto para o feto. As gestantes também são mais propensas a desenvolver tempestades de citocinas, indicativas de infecção viral grave, que podem induzir a vasoconstrição e a restrição do crescimento fetal intrauterino^(18)^.

Das condições crônicas pré-existentes entre as gestantes desse estudo, as mais comuns foram o diabetes, as doenças cardiovasculares e a obesidade. Isso pode estar relacionado com a alteração das atividades de vida diária, devido aos quadros de *lockdown* estabelecidos pelos órgãos públicos, interrupção do pré-natal de rotina e gerenciamento das condições de saúde em serviços de saúde, e aumento do sedentarismo e ganho de peso excessivo nos primeiros anos da pandemia de COVID-19. Além disso, as taxas de diabetes gestacional também aumentaram entre os anos de 2020 e 2021 em diferentes países do mundo^(19)^.

Em gestantes, tanto a obesidade como o diabetes elevam as citocinas pró-inflamatórias e adipocinas, como TNF-*α*, IL-6, IL-1*β*, leptina e resistina envolvidas na resposta inflamatória. Ademais, a própria resistência insulínica gerada pelas duas enfermidades supracitadas pode afetar o funcionamento de células do sistema imune e contribuir para reduzir a eficácia de vacinas nessa população^(20)^. Para a amostra estudada, isto pode ter comprometido o grau de sobrevida, considerando a baixa taxa de mulheres com esquema vacinal completo.

Para além da associação encontrada entre a COVID-19 e o diabetes (p = 0,023), destaca-se também a associação entre o uso e abuso de substâncias (p = 0,001). Estudo canadense mostrou que sintomas mais fortes de depressão e dificuldades financeiras foram associados ao aumento do uso e abuso de substâncias na gravidez^(21)^. Por sua vez, outro estudo norte-americano mostrou que problemas de saúde mental/emocional pré-pandemia de COVID-19 foram significantemente associados a um maior uso e abuso de substâncias para lidar com o estresse e sintomas depressivos causados pela pandemia. Isso maximiza alterações no sistema nervoso central e pode gerar riscos como a dependência de substâncias nocivas à saúde. Em altas concentrações, o uso de substâncias como cocaína e seus derivados durante a gravidez está ligado a altas concentrações de estrogênio, que modificam o funcionamento do sistema respiratório, levando ao aumento da vascularização local e maior absorção dessas drogas. Já no uso e abuso de tabaco, o acúmulo de nicotina pode atravessar a barreira placentária e comprometer a maturação fetal^(22)^. Nesse sentido, torna-se imperioso trabalhar discussões e intervenções que abordem a prevenção e o controle de estresse, ansiedade, depressão e comportamentos de risco com essa população.

Associações entre o diagnóstico de COVID-19 e doenças neurológicas (p = 0,001) estiveram presentes entre as gestantes. Evidências mostram que o acidente vascular cerebral isquêmico é a complicação neurológica mais comum da COVID-19, afetando até 6% de todos os pacientes com a infecção. Especificamente em gestantes, uma revisão de escopo identificou registros de alterações como delírio, encefalopatia, doença cerebrovascular e síndrome de Guillain-Barré associadas à COVID-19^(23)^. Por outro lado, estudo conduzido no nordeste brasileiro mostrou que não houve diferença na manifestação de complicações neurológicas ao comparar mulheres grávidas e não grávidas com COVID-19^(24)^. Todavia, os dados sobre o assunto ainda são escassos e carecem de maiores investigações.

As queixas clínicas mais comuns apresentadas pelas gestantes foram similares àquelas encontradas nas demais populações, sendo tosse, febre e dispneia os sintomas mais prevalentes. Estudo transversal iraniano também mostrou que tosse e dispneia foram os mais comuns entre as mulheres com o vírus SARS-CoV-2, elevando a demanda de oxigenoterapia^(25)^. Nesse estudo, o suporte ventilatório foi requerido por 28,3% das gestantes, e quase 10% delas necessitaram de ventilação invasiva. Dados de uma pesquisa conduzida na Ásia mostraram que a taxa de suporte de oxigênio de baixo fluxo foi de 52,7% entre as gestantes hospitalizadas, e que 2,7% da população necessitou ser intubada^(25)^.

Na maioria dos casos, a administração de oxigenoterapia e o manejo de alterações respiratórias graves é realizada em unidades de terapia intensiva. Nessa pesquisa, 21,3% das mulheres estavam internadas nessas unidades. Esses resultados foram menores que os de uma pesquisa iraniana, em que a taxa de internação foi 36%^(25)^. Uma revisão sistemática que investigou complicações maternas e neonatais por COVID-19 identificou que a admissão das gestantes em unidades de terapia intensiva foi comum na maioria das pesquisas sobre o tema, com uma taxa de ocorrência de 52,9%. Para mais, outros desfechos negativos identificados foram parto prematuro, morte materna, ruptura prematura das membranas, pré-eclâmpsia, crescimento intrauterino restrito e bebê natimorto^(26)^. Isso demonstra a importância de cuidados pré-natais sistematizados, sob um olhar atento de profissionais que lançam mão de práticas avançadas na sua assistência em saúde, como os enfermeiros.

Nessa pesquisa, a mortalidade materna foi de 7%. Estudo brasileiro que analisou os primeiros dois anos da pandemia por COVID-19 mostrou que a taxa de mortalidade aumentou em duas vezes (7,7 para 15,4%) entre a primeira (2020) e a segunda (2021) onda de casos da doença. Os índices foram ainda maiores em mulheres com comorbidades^(27)^. Dados semelhantes também foram encontrados na Índia^(28)^. O tempo de diagnóstico da doença também chama atenção. Segundo a análise realizada, foram precisos 42 dias para que houvesse a confirmação do caso. Isso foi influenciado pela existência de comorbidades, cor da pele das gestantes e região do país. O atraso na conclusão epidemiológica em mulheres com múltiplas condições de saúde pode ser relacionado ao fato da maior complexidade na condução dos cuidados e nos diagnósticos diferenciais arrolados. Em relação à cor da pele das gestantes, resultados de outra pesquisa brasileira mostram que o racismo pode ser o grande desencadeador de desfechos negativos. A letalidade por COVID-19 em mulheres brancas foi 14% menor em comparação com mulheres negras e pardas. No puerpério, a probabilidade de morte de mulheres negras foi 62% maior^(29)^.

Essas condições evidenciam a necessidade da identificação de determinantes sociais de saúde na construção e condução dos cuidados voltados à promoção da saúde, prevenção de doenças e agravos e no gerenciamento de alterações clínicas e enfermidades crônicas associadas, sobretudo em populações de risco, como as gestantes. Frente a isso, ressalta-se o protagonismo dos profissionais de enfermagem no âmbito da atenção primária à saúde e no contexto hospitalar, com cuidados críticos voltados ao binômino mãe-bebê. Para mais, a inserção de enfermeiros em sítios de vigilância epidemiológica deve ser estimulada, tal qual um maior aprofundamento acerca do zelo nos processos de identificação, notificação e divulgação dos dados.

Embora nosso estudo tenha produzido uma melhor compreensão sobre a associação das condições crônicas de saúde com o diagnóstico de SRAG em gestantes, exige-se uma interpretação cautelosa sobre os achados, e algumas limitações precisam ser evidenciadas. Nesse estudo, não foi possível levantar indicadores clínicos e laboratoriais específicos sobre as condições de saúde apresentadas. Informações sobre uso e abuso de substâncias foram baseadas no autorrelato das gestantes, o que pode ter levado a potenciais omissões nas respostas. A identificação acerca das razões para o atraso no diagnóstico dos casos de SRAG nas mulheres grávidas e os fatores de influência também foram algumas das limitações encontradas. Apesar dessas deficiências, é possível depreender que os resultados apresentados são valiosos para o direcionamento assistencial da enfermagem, bem como de outras áreas, e significativos para a elaboração de políticas públicas de saúde voltadas à população de interesse.

## CONCLUSÃO

A presença prévia de diabetes mellitus, doença neurológica e uso e abuso de substâncias esteve envolvida no diagnóstico, mas não na morte de gestantes com SRAG. Outras investigações utilizando dados de base populacional precisam ser desenhadas para aclarar os fatores de influência nos processos de adoecimento e mortalidade de gestantes e puérperas.
